# The Impact of Household Migration on the Intergenerational Educational Mobility: Based on the Perspective of Adolescent Development

**DOI:** 10.3390/ijerph20064825

**Published:** 2023-03-09

**Authors:** Xiaohe Zhou, Mingda Cheng, Chunhui Ye

**Affiliations:** China Academy for Rural Development, Zhejiang University, Hangzhou 310058, China

**Keywords:** household migration, intergenerational educational mobility, adolescent development

## Abstract

Improving intergenerational mobility is crucial for enhancing the efficacy of human capital, ensuring social vitality, and supporting sustainable long-term economic growth. Based on the China Labor-force Dynamic Survey (CLDS) of 2014, this paper empirically examines the effect of adolescent household migration on intergenerational educational mobility by using a fixed-effect model. The study found that: (1) Household migration in the adolescent period significantly improves intergenerational educational mobility. (2) The quality and quantity of education of offspring are the channels through which household migration improves the intergenerational educational mobility of the household. (3) There are significant differences between urban and rural areas, gender, and household resource allocation in the effect of adolescent household migration on intergenerational educational mobility. As the majority of poor households are unable to improve intergenerational mobility through migration due to its costs and institutional barriers, this paper suggests that the government should concentrate on reducing regional disparities in educational resources, advancing rural education reform, and enhancing social security.

## 1. Introduction

As it relates to a society’s long-term fairness and efficacy, social mobility is a crucial aspect of development and public research [1]. Research has demonstrated that greater social mobility has a positive effect on class mobility, and studies have shown that intergenerational mobility in China continues to weaken [2,3,4]. Therefore, how to effectively improve intergenerational social mobility and weaken class barriers is of great significance to maintaining social harmony and stability, maintaining social and economic vitality, and promoting benign social operation and coordinated development.

Migration has always been one of the feasible channels for Chinese residents to improve their intergenerational mobility. As early as the Warring States Period (475–221 BC), the mother of Mencius relocated multiple times in order to find a suitable environment for her son, giving rise to the urban legend “Mencius’ mother moves three times.” Individual development is closely related to the geographical environment. Residents of poor areas are unable to realize their full potential due to the limitations of their geographical environment and supporting facilities. In the meantime, the nature of intergenerational transmission of poverty will lead to a gradual decrease in the intergenerational mobility of families. Consequently, migration to better areas has become a viable option for them to increase intergenerational mobility.

Since China’s reform and opening up in 1978, with the continuous development of the economy, the gradual liberalization of the household registration system, and the gradual improvement of transportation facilities, a large number of poor rural people in remote areas have migrated to urban areas for decent wages, better living environment [5] and fair competition opportunities [6]. To date, the majority of existing research has confirmed the positive effect of its migration behavior on intergenerational mobility. However, most of the existing studies only explored the impact of personal migration on the intergenerational mobility of families after adulthood [7,8,9]. While there is little literature on the impact of household migration on intergenerational mobility from the perspective of adolescent development, which is due to variations in China’s national conditions throughout time. Given the strict restrictions of the early Chinese household registration system and the low degree of urbanization, migrants are not permitted to relocate their families as a unit, and the majority of migrants are individual migrant workers who work in urban areas without altering their rural household registration position. Consequently, previous research has focused more on the impact of individual migration on intergenerational mobility [6,9,10,11,12,13]. For example, Sun et al. [6] used the Heckman two-stage method to find the impact of individual migration on the intergenerational elasticity of household income by taking the local migration probability as a tool variable. Li and Li [11] used China Family Panel Studies (CFPS) data to examine the impact of migration on intergenerational mobility and found that individual migration can significantly improve intergenerational income mobility. Wald [9] studied the economic returns brought by migration by making use of the difference between brothers’ migration and not and found that individual migration significantly improved economic status and significantly increased intergenerational income mobility. Later, with the extension of migrant workers’ living time in cities and the improvement of urbanization level, the migration of the labor force continues to promote household migration, which has gradually become the main trend of population migration in China [14,15], while the current research on the impact of household migration on intergenerational mobility is insufficient.

Migration is a household decision-making behavior, which will not only affect the career planning and social network of adult families but also affect the quality of education and character development of teenagers. Research shows that the personal growth environment affects the development of personal characteristics [16,17,18,19,20,21]. Household migration to a better environment helps adolescents’ cognitive development and human capital accumulation [22,23,24,25], and the impact of household migration on intergenerational mobility will be reflected more in the cultivation of household adolescents as young people are more malleable and susceptible to environmental changes. Therefore, by changing the growth environment of young people, household migration may affect the development of young people, thus affecting the intergenerational mobility of families.

Currently, many scholars have studied the impact of migration on intergenerational mobility from the perspectives of developed and developing countries, but no consistent conclusions have been drawn. On the one hand, some scholars evaluated the effect of migration programs in developed countries, for example, the Moving to Opportunity (MTO) experiment, a large-scale migration project that ran in the United States from 1994 to 1998 [24,26,27]. The aim of MTO was to provide housing vouchers to families, giving low-income families the opportunity to move from high-poverty areas to low-poverty areas, thus alleviating poverty by improving living environments. Analysis of the MTO experiment evaluation shows that people who moved to “high-opportunity areas” during childhood could achieve higher incomes when they became adults, indicating that the community in which children grow up can, to some extent, influence intergenerational mobility in society. On the other hand, some scholars have explored the impact of migration on intergenerational mobility in developing countries. For example, Barnhardt et al. [28] found in their study of India that providing housing subsidies to poor families and moving them to nearby urban commercial areas resulted in a significant decrease in the human capital and income levels of their adult children, indicating that intergenerational mobility in their families was actually reduced. Overall, there have been fewer studies on the impact of migration on intergenerational mobility in developing countries. Moreover, because the conclusions for developed and developing countries are inconsistent, the evaluation experience of developed countries cannot be fully applied to developing countries. Therefore, evaluating the impact of migration on intergenerational mobility in developing countries is very important in supplementing existing research deficiencies.

Existing studies have measured intergenerational mobility mainly from three aspects: income, occupation, and education [29,30,31,32,33]. However, this paper focuses on intergenerational educational mobility based on two considerations. First, as the most important component of human capital, the improvement of education level has a significant positive impact on an individual’s future income level and occupation position. Previous studies have found that improving intergenerational mobility in education can enhance both income and occupational intergenerational mobility [34,35]. Thus, it is difficult to separate the role of education to evaluate the pure impact of migration on income and occupation. Second, the effect of migration on adolescents is mainly to change the environment. Individuals’ educational level is more susceptible to their early environmental exposure. Differences in teaching quality and educational environment directly affect the level of education acquired and the intergenerational mobility of families [36,37]. However, intergenerational mobility of income and occupation are more likely to be influenced by factors such as household background and social capital, in addition to the impact of the educational environment.

On the basis of the above literature review and potential research gap, this paper takes the intergenerational educational mobility of households as the research object has three main research goals: First, verifying whether the adolescence period of household migration has a significant positive impact on the improvement of intergenerational educational mobility. Second, exploring the channels through which household migration in adolescence could affect intergenerational educational mobility. Third, expanding the heterogeneity analysis from the perspective of location, gender differences, and resource constraints.

Using a nationwide household survey dataset covering unique migration information from the China Labor-force Dynamic Survey (CLDS) of 2014, this paper empirically evaluated the effect of adolescent household migration on intergenerational educational mobility. By adopting a fixed-effect model, this study found that household migration in the adolescent period significantly improves intergenerational educational mobility in China. The results of mechanism analysis showed that more opportunities to receive better education for the migrated offspring is a key factor in improving intergenerational mobility. We also confirmed the heterogeneity effects bought by the gender and location differences.

The paper is organized as follows. Section 2 describes the data sources and reports summary statistics. Section 3 presents empirical strategies and econometric models. Section 4 presents empirical results and analysis, and Section 5 concludes.

## 2. Data

Given the main research content of this paper, the sample data adopted in this paper should comprehensively cover the information on household migration, education levels of parents and their children, and relevant individual and characteristic household variables. Based on the above requirements, this paper used the individual survey database of the China Labor Force Dynamics Survey (CLDS) in 2014. It is the first national follow-up survey focusing on labor migration in China. The sample size of this paper is 23,594 observations, which are distributed in 29 provinces, municipalities, and autonomous regions of China (except Hong Kong, Macao, Taiwan, Tibet, and Hainan). These data provides detailed information about household migration, which makes it possible to identify the impact of household migration on intergenerational educational mobility.

This study focuses on the impact of adolescent household migration on intergenerational educational mobility. There are two core variables, namely, intergenerational educational mobility and adolescent household migration. First, regarding the measurement of intergenerational educational mobility, this paper refers to the measurement of Liang and Li [38] and uses the education level gap between parents and their offspring as the measurement of intergenerational educational mobility of the household. The larger the difference, the better the household has intergenerational educational mobility. Secondly, for the measurement of household migration, we strictly follow the academic definition of “migration” based on “hukou change” [39,40]. According to the CLDS questionnaire, people should answer “whether your household registration has moved” and “in which year your household registration has moved.” Thus, we could comprehensively determine whether a person has migrated with their household during his teenage years and form the indicator of household migration.

According to the existing literature, the influencing factors of adolescents’ education level and intergenerational mobility of household include individual characteristics and household characteristics, which should also be controlled in our empirical model. Specifically, the control variables of individual characteristics include age, gender, and political background. The control variables of household characteristics include household income, urban or rural registration, and household member. In addition, we use the quantity and quality of children’s education as channels through which household migration affects intergenerational educational mobility. Table 1 reports the definitions and descriptions of the variables required for empirical research.

## 3. Empirical Strategies and Econometric Models

This paper has the following three research goals: First, as mentioned in the introduction, the main purpose of this paper is to verify whether the adolescence period of household migration has a significant positive impact on the improvement of intergenerational educational mobility. We use the following model (1) to test the hypothesis.

(1) $$Y_{ikc}-Y_{jkc}=\alpha +\beta Treat_{k}+X_{i}D+v_{k}E+\varphi_{c}+\varepsilon_{ijc}$$

where $Y_{ikc}$ is the education level of individual *i* in household *k* city *c*. $Y_{jkc}$ is the education level of individual *j* in household *k* city *c*, where individual *j* and individual *i* are parent-child relationship. So $Y_{ikc}-Y_{jkc}$ represents educational intergenerational mobility. $Treat_{k}$ indicates whether the household *k* has migrated during individual *i*’s adolescence period. Therefore, *β* is the estimated coefficient of the impact of household migration on intergenerational educational mobility. $X_{i}$ are the individual control variables. $v_{k}$ are the household control variables. $\varphi_{c}$ is the city fixed effect, $\varepsilon_{ijc}$ represents the random error term of the regression equation.

Regarding the endogeneity of the model, studies have shown that migration is a highly selective process [41], so there may be a systematic gap between migrating families and non-migrating families. However, the migration subject studied in this paper is migration during the adolescent period. As the passive recipient of migration decisions, whether they migrate in their adolescence will not be affected by their future adult education level. Thus, avoiding the problem of bidirectional causality. In addition, individual-level, household-level control variables, Hukou, and city-fixed effects are also controlled in the empirical model to reduce the possible bias caused by omitted variables. Therefore, model (1) has a low possibility of regression bias due to the endogeneity problem.

Second, based on the regression results model (1), we further explore the channel through which household migration in adolescence could improve intergenerational educational mobility. As described in the model (1), the education level gap between individuals and their father represents intergenerational educational mobility. Since fathers’ education level can hardly be changed when the household migrated in the adolescence of children, the improvement of intergenerational educational mobility of the household is mainly due to the improvement of the education level of their offspring. Numerous studies have shown that the adolescent period is the golden period for character building and human capital cultivation, and moving to a better area is conducive to the cognitive development and human capital accumulation of adolescents [22,23]. Thus, this paper uses model (2) to explore how household migration in adolescence affects intergenerational educational mobility from the perspective of offspring’s education quantity and education quality.

(2) $$Y_{ikc}{}^{q}=\alpha +\beta Treat_{k}+X_{i}D+v_{k}E+\varphi_{c}+\varepsilon_{ic}$$

$Y_{ikc}{}^{q}$ is the quality or quantity of education that individual *i* received when he was in his adolescence. More specifically, the education quantity evaluates whether the adolescent of migrant households have higher education when they become adults, and the education quality evaluates whether the adolescent obtains better educational resources during their studies. $Treat_{k}$ indicates whether the *k* household has moved during *i*’s adolescence. Therefore, *β* is the estimated coefficient of the impact of household migration on the quality or quantity of education. $X_{i}$ is the individual control variable of the offspring. $v_{k}$ is the household control variable. In addition, $\varphi_{c}$ is the city fixed effect. $\varepsilon_{ic}$ represents the random error term of the regression equation.

Lastly, in view of the serious inequality of educational opportunities between urban and rural areas [42,43], traditional gender discrimination [44], education resources compatriots crowding effect under the household constraints [45], this further paper studies the heterogeneity effects of household migration in hukou location, gender discrimination and number of siblings during adolescence on the intergenerational educational mobility. Specifically, based on the above empirical model (1), the empirical results are obtained in the form of sub-sample regression.

## 4. Empirical Analysis

### 4.1. Effect of Adolescence Migration on Intergenerational Educational Mobility

Table 2 shows the outcomes of estimating Equation (1). Columns (1) to (3) present estimation results where we incrementally include sets of covariates and fixed effects, starting with a basic model with only controls in column (1) to the full model in column (3). As all three models deliver similar results and inferences, the impact of adolescent household migration on intergenerational educational mobility is significantly positive at the 1% significance level (*p* < 0.01). We focus our discussion on the full specification model. According to the estimation result of column (3), it indicates that when a household migrated during the adolescence of its offspring, the increasing years of education by offspring compared to their father are 0.445 years larger than those of the household without migration, proving adolescence period of household migration has a significant positive impact on the improvement of intergenerational educational mobility.

### 4.2. Mechanism Analysis Based on the Quality and Quantity of Adolescent Education

On the basis of testing in Section 4.1, we further test research goal 2 by conducting a regression (Equation (2)) to explain how household migration has a positive impact on intergenerational educational mobility from the education quantity and education quality of the offspring. As can be seen in Table 3, the dependent variable in columns (1)–(3) is the quantity of education which indicates the years of education of the offspring. The dependent variable in columns (4)–(6) is quality of education which indicates whether the offspring ever studied in key middle schools or key universities. Consistent with Table 2, regardless of whether control variables are added, or fixed effects are removed, the coefficients are significantly positive at the 1% significance level (*p* < 0.01) throughout columns (1)–(6), indicating that the results remain robust and consistent.

Similar to Table 2, we focus our discussion on the full specification model in columns (3) and (6). If the individuals migrated with their household during their adolescence, their years of education would increase by 1.099 years compared with those individuals who did not migrate during their adolescence. In addition, they were also 4.8% more likely to attend a key high school or university than those who did not migrate. This result is consistent with Nakamura et al. [46], who demonstrated that household migration significantly improves the lifelong income and educational attainment of children within the family, using the eruption of a volcano in Iceland in 1973 as a natural experiment. In combination with the conclusion of this study, it suggests that household migration during their offspring’s adolescence may provide more opportunities for migrated adolescents to receive higher-quality education due to better infrastructure and educational resources. For example, they have a higher probability of entering key middle schools or key universities when they are studying, and they will also have a higher education level in the future. Therefore, under the condition that the father’s education level remains unchanged, the household migration would ultimately improve the intergenerational educational mobility by improving the offspring’s education level and quality.

### 4.3. Heterogeneity Analysis

This section explores the heterogeneous impact of adolescent household migration on intergenerational educational mobility of households from three aspects: urban-rural differences, gender differences, and resource allocation differences.

First, since the reform and opening-up policy, with the rapid advancement of industrialization in China’s coastal cities and the gradual relaxation of restrictions on the flow of rural labor, urbanization in China has developed rapidly. According to the data provided by the seventh national population census of China in 2020, the urbanization rate in China was 63.89% in 2020, an increase in 14.21 percentage points compared to 2010 (See more detail at http://www.gov.cn/guoqing/2021-05/13/content_5606149.htm (accessed on 1 March 2023)). While urbanization can promote economic growth, too rapid urbanization can also bring hidden dangers of unbalanced urban-rural development, among which the further widening of the gap in educational resources between urban and rural areas is one of the main manifestations. Research has shown that there has always been a serious inequality of educational opportunities between urban and rural areas in China [47]. Educational funds and high-quality educational resources are more inclined to urban rather than rural areas. According to the existing literature, the main population flow in China is the migration of the surplus rural labor force to cities, so the improvement of intergenerational educational mobility may mainly come from the migration of households from rural areas to cities, thus reducing the impact of the inequality of education resources in rural areas on themselves. In this regard, the samples in Table 4 are divided into rural areas and urban areas according to their migration destinations for grouping regression. According to the regression results, the intergenerational educational mobility of households migrating to rural areas did not significantly improve, while the intergenerational educational mobility of households migrating to urban areas increased by 0.735 years at the significance level of 1%. The above differences in the regression results of urban and rural sub-samples indicate that migration improves intergenerational household mobility by improving access to educational resources for offspring, and only migration to urban areas with better educational resources can significantly improve intergenerational mobility.

Secondly, there has always been a preference for sons over daughters in Chinese families. According to data provided by the 2020 “China Population Census Yearbook,” the proportion of illiterate people aged 15 and above 15 in China was 4.95% for females, nearly three times that of males at 1.62%. This gender disparity leads to a more severe inequality in educational opportunities in rural areas, where the proportion of illiterate women aged 15 and above is 9.07%, significantly higher than the 2.98% for men (See more detail at http://www.stats.gov.cn/tjsj/pcsj/rkpc/7rp/zk/indexce.htm (accessed on 6 March 2023)). In recent years, although the gender gap in education has been narrowed to a certain extent, women still lag behind men in terms of higher education access opportunities, dropout risk, education quality, labor market returns, and various indicators of educational outcomes, sex differences in education is still an important realistic problem [44]. Therefore, we need to verify whether there are still gender differences in the improvement of intergenerational educational mobility by adolescent household migration. From columns (4) and (5) of Table 4, both male and female offspring of household migration improve intergenerational educational mobility. However, males gained 0.809 years of intergenerational educational mobility at a 1% significance level, while females gained only 0.359 years at a 10% significance level. Therefore, whether from the significance level or coefficient, families with female offspring have lower migration benefits than those with male offspring, which proves that gender differences still exist.

In addition, we try to explain the gender difference in the impact of household migration on intergenerational educational mobility through sibling crowding of educational resources. Due to the resource-crowding effect of compatriots in education [48], under the constraints of household resources, the more children in a household, the fewer educational resources each child will get. That is, parents will selectively allocate educational resources. Combined with the concept of favoring boys over girls in China, a household with multiple children is more inclined to concentrate educational resources on male offspring under resource constraints, thus affecting gender differences. Table 5 shows the heterogeneous regression results of the sibling crowding effect. In the paper, the number of children in a household is based on whether the number of siblings exceeds the median. Panel A is the sample with less than the median number of siblings, indicating that they are less constrained by household resources. Panel B is the sample with more siblings than the median, indicating that they are more constrained by household resources. From Table 5, in families less constrained by resources, there are still gender differences in the impact of household migration on intergenerational educational mobility. However, compared with the difference in columns (4) and (5) in Table 4, the sample difference in panel A is smaller, and the female is still significantly positive. As for panel B, in families with more resource constraints, it can be found that the impact of household migration on intergenerational educational mobility is still significant when the offspring is male but not when the offspring are female. The regression results confirm the view that due to the Chinese preference for boys and the constraints of household resources, parents tend to allocate more limited educational resources to male offspring when household resources are limited, which further aggravates gender inequality in educational access.

### 4.4. Robustness Checks

In order to further test the robustness and consistency of the benchmark regression conclusion, this paper adopted the following measures for the robustness test. Firstly, adjust the city fixed effect to the provincial fixed effect, and show the results in column (1). Secondly, change the clustering level, adjust the clustering level of the city to the provincial level or individual level, and display it in columns (2)–(3). Then, the alternative independent variables are replaced, and the measurement of intergenerational educational mobility in the original regression model is represented by the difference between the education level of the offspring and the father. In column (4), the index of intergenerational educational mobility is replaced by gender. Specifically, the index measure of intergenerational educational mobility of a household with male offspring is the difference between the education level of a son and his father, while that of a household with female offspring is the difference between the education level of a daughter and her mother. In column (5), the parent’s highest education level is used to replace the father’s education level, and the measure of intergenerational educational mobility is replaced with this condition for regression. Finally, in column (6), one concern regarding the measurement of the cause variable is that the absolute difference in educational attainment between the two generations does not effectively reflect the distribution of education among their respective cohorts. To address this concern, we refer to the measurement of Bukodi and Goldthorpe [49], who adopt relative educational differences to measure intergenerational educational mobility. Specifically, we divide both parent and child generations into five groups according to the level of education and obtain their respective relative education levels. We then subtract the relative education levels of the two generations to generate the relative differences in education levels. Since this method estimates the differences in relative educational status between two generations, it partially circumvents the issue of the original measurement indicator being unable to reflect the educational distribution of their respective birth cohorts. The results of correlation regression are shown in Table 6. The regression results of the three robustness tests above are significantly positive at the 1% significance level, and there is no significant difference between their regression coefficients and the benchmark regression results in Table 2, which indicates that the original model and regression results are robust and reliable.

### 4.5. Other Threats to Identifications

As mentioned in the introduction, migration is a highly self-selective process that is influenced by various regional and family factors. Therefore, we need to eliminate the interference of other factors to ensure that our benchmark results are robust.

First, offspring in different birth cohorts are exposed to different educational environments and educational policies. Therefore, to remove the interference bought by birth cohorts, we generated a new variable that divides offspring birth order into four birth cohorts (before 1970, 1970–1980, 1980–1990, and after 1990), and then we added the fixed effects of this variable in Table 7 column (2) to ensure that the intergenerational mobility of different families is compared within the same birth cohort of offspring.

Second, the choices of household migration are closely related to regional characteristics, and the lack of controlling those variables can cause endogeneity problems. Basically, there are two aspects of regional characteristics that may affect the choice of migration: the regional characteristics of the city where they were located before migration and the regional characteristics of the migration destination. As can be seen from the econometric model in Section 3, we have controlled the city-fixed effects, which removed all unobservable effects at the city level of migration destinations. However, we could not accurately control for the regional characteristics of the city before migration as we lacked information on where the households were located before the migration. To compensate for this shortcoming, we control for respondent birthplace fixed effects in column (3).

We used the baseline result in column (1) as a comparison. We found that, whether adding birth order fixed effects in column (2) or birthplace fixed effects in column (3), the regression coefficients and significance remained unchanged compared to column (1), indicating the robustness of the baseline results after controlling for other factors.

## 5. Conclusions

Improving intergenerational mobility is of great significance to improving the efficiency of human capital, ensuring social vitality, and promoting long-term stable economic growth. Based on the 2014 CLDS database, this paper empirically examines the effect of adolescent household migration on intergenerational educational mobility by using a fixed-effect model and explores its channels and heterogeneity results.

The results of the study are as follows. (1) Adolescent household migration significantly improves intergenerational educational mobility. Compared with households that did not migrate, the offspring of households that migrated experienced greater educational improvement than their parents. (2) The quality and quantity of education of offspring are the channels through which household migration improves the intergenerational educational mobility of the household. Due to access to better educational resources and a teaching environment, the offspring of migrant households have a higher probability of entering key middle schools or key universities and will have a higher number of years of education. (3) There are significant differences between urban and rural areas, gender, and household resource allocation in the effect of adolescent household migration on intergenerational educational mobility. First of all, intergenerational educational mobility is significantly improved only for household moving to urban areas but not for household moving to rural areas. In addition, the income of male offspring in household migration is much higher than that of female offspring. Finally, the sibling crowding effect of resource allocation exists more in female offspring, which further aggravates gender inequality in education access.

The above research results show that adolescent household migration does contribute to the improvement of intergenerational educational mobility, but the channel of improvement is due to the inequality of educational resources between regions, that is, the better educational resources and educational environment of the migrating region enable the offspring to obtain a better education. Moreover, there are serious differences between urban and rural areas and gender discrimination in the effect of migration on intergenerational educational mobility, which also reflects the difference in educational resources between urban and rural areas and the disadvantageous position of the female in education access.

Accordingly, this paper proposes the following policy implications based on two perspectives: that of the Chinese government and that of other developing countries around the world. On the one hand, to improve educational intergenerational educational mobility in China. Firstly, the government should be committed to alleviating the differences in educational resources between regions, which is the main reason why migration can improve intergenerational educational mobility. Due to migration costs and institutional barriers, most households cannot improve intergenerational mobility through migration. Therefore, the Chinese government should increase financial support for education in areas with weak educational resources and make overall planning of educational resources in different regions to maintain a balance so that households unable to migrate can also obtain high-quality educational resources. Secondly, the government should intensify efforts to deepen the reform of rural education and provide more educational subsidies and resources for rural and backward areas so that they can have more opportunities to receive high-quality educational resources. Thirdly, local governments should provide a better social security system, ease budgetary constraints on education for rural and poor families, and improve the availability of educational resources for females. In addition, the government should strengthen the publicity of gender equality and alleviate the preference for sons in rural and underdeveloped areas.

On the other hand, this article is one of the few that evaluates the impact of migration on intergenerational household mobility in developing countries. In recent years, a growing number of developing countries, drawing on the successful experience of migration programs in developing countries that have improved intergenerational mobility, have implemented costly programs to improve intergenerational mobility for the poor. However, previous research in developing countries suggests that the potential effects of migration on enhancing intergenerational mobility do not match those of developed countries. Through mechanistic and rural-urban heterogeneity analysis, our paper demonstrates that the improvement in intergenerational mobility from migration is due to the better environment and more opportunities for the migration destination. This, in turn, explains the failure of migration programs in some developing nations, which provided poorer public services, fewer interpersonal networks, and lower migration benefits in the selected migration destinations. Therefore, the findings of our paper have significant implications for enhancing intergenerational mobility in other developing countries.

## Figures and Tables

**Table 1:** Descriptive statistical analysis.

Variables	Obs.	Definition	Unit	Mean	S.D.	Min	Max
**Migration variables**							
Household migration	20,723	Whether migrated with household before adulthood No = 0, Yes = 1	(0,1)	0.055	0.228	0	1
**Intergenerational mobility**							
Quantity of education	23,527	Individuals years of education	years	8.576	4.435	0	22
Quality of education	23,527	Whether once studied in key middle school or key university 0 = No 1 = Yes	(0,1)	0.074	0.262	0	1
Mother’s education level	22,277	Mother’s years of education	years	3.607	4.243	0	19
Father’s education level	22,006	Father’s years of education	years	5.059	4.536	0	22
Intergenerational educational mobility	21,970	(Individuals’ Education level-Father’s education level)	years	3.580	4.397	−16	22
**Other variables**							
Gender	23,593	Male = 0, female = 1	(0,1)	0.520	0.500	0	1
Age	23,439	Age	years	43.88	14.50	14	114
Hukou	23,594	Rural = 0, Urban = 1	(0,1)	0.388	0.487	0	1
Income	23,030	Household income	1000 CNY/yr	58.64	117.2	0	6000
Household member	23,594	Number of people in the same household registration book	Number	4.533	1.980	1	20
Number of siblings	23,558	Number of siblings in household	Number	3.023	2.033	0	15

**Table 2:** Effect of adolescence migration on intergenerational educational mobility.

Variables	Intergenerational Educational Mobility		
	(1)	(2)	(3)
Migration	0.519 ***	0.328 **	0.445 ***
	(0.141)	(0.141)	(0.150)
Observations	18,836	18,836	18,836
Control	YES	YES	YES
Hukou FE	NO	YES	YES
City FE	NO	NO	YES

Note: ***, ** represent statistical significance at 1% and 5% probability levels, respectively. Standard errors are clustered at the city level. Control variables include gender, age, household income, and household members. Hukou FE indicates whether the household lives in rural or urban areas.

**Table 3:** Mechanism analysis based on the quality and quantity of adolescent education.

Variables	Quantity of Education			Quality of Education		
	(1)	(2)	(3)	(4)	(5)	(6)
Migration	1.967 ***	1.265 ***	1.099 ***	0.052 ***	0.046 ***	0.048 ***
	(0.203)	(0.174)	(0.146)	(0.014)	(0.013)	(0.012)
Observations	20,042	20,042	20,042	20,660	20,042	20,042
Control	YES	YES	YES	YES	YES	YES
Hukou FE	NO	YES	YES	NO	YES	YES
City FE	NO	NO	YES	NO	NO	YES

Note: *** represents statistical significance at 1% probability level, respectively. Standard errors are clustered at the city level. Control variables include gender, age, marital status, household income, and household members. Hukou indicates whether the household lives in rural or urban areas.

**Table 4:** Urban-rural differences and gender differences.

Variables	Intergenerational Educational Mobility				
	(1)	(2)	(3)	(4)	(5)
	All	Rural	Urban	Male	Female
Migration	0.445 ***	0.073	0.735 ***	0.809 ***	0.359 *
	(0.150)	(0.243)	(0.209)	(0.255)	(0.189)
Observations	18,836	11,687	7149	9838	8998
Control	YES	YES	YES	YES	YES
Hukou FE	YES	YES	YES	YES	YES
City FE	YES	YES	YES	YES	YES

Note: *** *p* < 0.01, * *p* < 0.1. Standard errors are clustered at the city level. Control variables include gender, age, hukou, marital status, household income, and household members. Hukou indicates whether the household lives in rural or urban areas.

**Table 5:** Sibling crowding effect.

Variables	Intergenerational Educational Mobility	
	(1)	(2)
	Male	Female
Panel A: Number of siblings < Median		
Migration	0.813 ***	0.553 *
	(0.291)	(0.302)
Observations	4752	3953
Panel B: Number of siblings ≥ Median		
Migration	1.036 **	0.327
	(0.414)	(0.260)
Observations	5086	5045
Control	YES	YES
Hukou FE	YES	YES
City FE	YES	YES

Note: *** *p* < 0.01, ** *p* < 0.05, * *p* < 0.1. Standard errors are clustered at the city level. Control variables include gender, age, hukou, marital status, household income, and household members. Hukou indicates whether the household lives in rural or urban areas.

**Table 6:** Robustness checks.

	Intergenerational Educational Mobility					
	FE	Custer		Independent Variable		
	Provincial	Provincial	Individual	Gender	Highest Education Level	Relative Educational Level
	(1)	(2)	(3)	(4)	(5)	(6)
Migration	0.447 ***	0.445 ***	0.445 ***	0.416 ***	0.474 ***	0.240 ***
	(0.164)	(0.160)	(0.146)	(0.151)	(0.148)	(0.052)
Observations	18,836	18,836	18,836	18,990	19,378	18,836
Control	YES	YES	YES	YES	YES	YES
Hukou FE	YES	YES	YES	YES	YES	YES
City FE	YES	YES	YES	YES	YES	YES

Note: *** *p* < 0.01. Standard errors are clustered at the province level in columns (1)–(2), individual level in column (3), and city level in columns (4)–(6). Control variables include gender, age, hukou, marital status, household income, and household members. Hukou indicates whether the household lives in rural or urban areas.

**Table 7:** Other threats to identifications.

Variables	Intergenerational Educational Mobility		
	(1)	(2)	(3)
Migration	0.445 ***	0.493 ***	0.423 ***
	(0.150)	(0.149)	(0.150)
Observations	18,836	18,836	18,462
Control	YES	YES	YES
Hukou FE	YES	YES	YES
City FE	YES	YES	YES

Note: *** *p* < 0.01. Standard errors are clustered at the city level. Control variables include gender, age, hukou, marital status, household income, and household members. Hukou indicates whether the household lives in rural or urban areas.

## Data Availability

China Labor-force Dynamic Survey (CLDS) can be obtained from the official website of the (Center of Social Survey, CSS) by the application at http://css.sysu.edu.cn (accessed on 1 March 2023). Email: cssdata@mail.sysu.edu.cn.
